# Association between media exposure and maternal health service use in Nepal: A further analysis of Nepal Demographic and Health Survey-2022

**DOI:** 10.1371/journal.pone.0297418

**Published:** 2024-03-11

**Authors:** Shreeman Sharma, Bikram Adhikari, Achyut Raj Pandey, Sulata Karki, Saugat Pratap K. C., Deepak Joshi, Sushil Chandra Baral

**Affiliations:** HERD International, Lalitpur, Nepal; Noble Shivapuri Research Institute, NEPAL

## Abstract

**Background:**

Nepal has made incremental progress in improving coverage of maternal health services leading to improved health outcomes. Government and other health sector stakeholders have consistently considered dissemination of educational messages on maternal health through mass media. However, in Nepal’s context, the media’s influence on the uptake of maternal health services is less known. This study examines the links between media exposure and maternal health service use in Nepal.

**Method:**

Our analysis is based on the nationally representative Nepal Demographic and Health Survey (NDHS) 2022 data. We analyzed data from 1933 women aged 15–49 who had given birth in the two years preceding the survey. Weight analysis was performed to account for complex survey design. We presented categorical variables as frequency, percentage, and corresponding 95% Confidence Interval (CI). Univariable and multivariable logistic regression assessed the association between media exposure and maternal health service use, and the results are presented as crude (COR) and adjusted odds ratios (AOR) along with 95% CI.

**Results:**

Women exposed to internet use had 1.59 times [AOR = 1.59, 95% CI = 1.16, 2.19], and those exposed to radio and television health programs had 1.73 times [AOR = 1.73, 95% CI = 1.17, 2.56] higher odds of having four or more Antenatal Care (ANC) visits. Similarly, women exposed to mass media had 1.32 times [AOR = 1.32, 95% CI = 1.00, 1.74] and those exposed to health programs had 1.50 times [AOR = 1.50, 95% CI = 1.02, 2.21] higher odds of having an institutional delivery. However, mass media exposure, internet use, and health program exposure were not significantly associated with increased postnatal care of mother and newborn.

**Conclusion:**

Exposure to health programs and internet use are positively associated with four or more ANC visits. Exposure to mass media and health programs are positively associated with increased institutional delivery. Our findings imply that well-designed campaigns and awareness programs delivered through mass media platforms play a vital role in enhancing the uptake of maternal health services.

## Introduction

Nepal has shown improving trends in maternal health outcomes over a decade. Compared to the 2011 Nepal Demographic Health Survey (NDHS), some indicators of maternal health, such as the rates of institutional delivery, Antenatal Care (ANC) visits and Postnatal Care (PNC) visits, showed incremental improvement in 2016 and 2022 [1, 2]. Despite this progress, disparities are persistent in maternal health service use, including ANC visits, institutional birth and PNC visits [3].

The evidence suggests that the media plays a key role in shaping health beliefs and behaviors, influencing health risk perception, disease prevention, maintenance of health and hygiene [4–6] Although general media content, such as day-to-day viewership of audiovisual or multimedia messages, may influence the audience, health programs involve audience-targeted media programs disseminated through radio and television [7]. Exposure to mass media and specific health programs’ impact on maternal health service use has been studied in various African and Asian countries [8–10]. Specific audience-targeted mass media campaigns and programs are found to have been more effective as they can, directly and indirectly, produce positive changes or prevent negative changes in health-related behaviors across large populations [11]. A 2014 study in Uganda showed a significant relationship between reading newspapers and being prepared for delivery among rural women regardless of the frequency of exposure [12]. Similarly, a 2016 study in Malawi revealed that women’s exposure to a community driven mass media campaign enhances the likelihood of seeking maternal healthcare services such as ANC and PNC than their unexposed counterparts [10].

Although the associations between media exposure and maternal health service use have been studied in different countries, studies are limited in Nepal that relate to the practices of watching television, listening to the radio, reading newspapers, using the internet and watching and listening to specific health programs by women and its impact on maternal health service use. According to the NDHS 2022, exposure to radio, newspaper, and television among women and men has decreased over time, with only 30% of women and 17% of men not being exposed to the three mass media in 2006, as compared with the population not being exposed to these mass media– 49% and 41%, respectively in 2022 [7]. Although the media has been considered an important route for propagating educational messaging, people’s exposure to specific types of media is inconsistent. In the context of the evolving nature of media and changing preferences of media use, this study has responded to the gap of the evidence on the association of mass media, internet and health programs of radio and television with maternal health service use. Based on the data sets of NDHS 2022, we have analyzed media exposure’s association with three indicators of the maternal health service use that includes ANC visits (4 times or more), institutional delivery, and PNC for mother and newborn. The existing evidence on the relationship between media exposure and maternal health service use provides the context before Nepal was federalized in 2015, and there is a lack of fresh evidence generated following these political and administrative reforms. Thus, the analysis of the nationally representative survey, first conducted after Nepal’s federalization and provincial restructuring, has presented latest evidence on the media exposure’s relation with maternal health service use in Nepal.

## Material and methods

### Study design

This study employed a secondary analysis of the NDHS 2022 dataset. The NDHS is a nationally representative survey that periodically updates the demographic and health information regarding the people of Nepal. The survey was implemented by the New ERA under the aegis of the Ministry of Health and Population (MoHP) and was technically supported by ICF International.

### Study setting

This study was conducted in Nepal, a landlocked South Asian country covering 147,181 km^2^ [13]. Since 2015, Nepal has been administratively divided into three tiers of government, including a federal government, seven provincial governments, and 753 local governments. Local government includes 6 metropolitan cities, 11 sub-metropolitan cities, 276 urban municipalities, and 460 rural municipalities. According to the National Population and Housing Census 2021, the total population of Nepal was 29,164,578, constituting 51.1% females and 48.9% males [14]. In 2020 the Human Development Index (HDI) of Nepal was 0.587, whereas the HDI of the rural and urban areas were 0.561 and 0.647 respectively, according to the Human Development Report of Nepal, 2020 [15].

### Sample and sampling

The NDHS 2022 samples are nationally representative, with all seven provinces of the country covered. First, stratification was done by dividing each province into urban and rural areas, which together constituted the sampling stratum for each province, leading to the formation of 14 sampling strata. The sampling was carried out in two stages. In the first stage, 476 primary sampling units (PSUs) were selected using probability-proportional-to-size, with 248 PSUs from urban areas and 228 from rural areas. The second stage involved selecting 30 households from each PSU, leading to a total sample size of 14,280 households, comprising 7,440 urban and 6,840 rural households. From among the surveyed households, 15,238 women aged 15–49 were eligible for NDHS survey, from which 14,845 were interviewed, resulting in a response rate of 97.42%. In this study, we analyzed the data of 1933 women aged 15–49 with a live birth in the two years preceding the survey to address our research questions.

### Data collection

The NDHS 2022 data were collected between 5^th^ January and 22^nd^ June 2022, by 19 teams. Each team was comprised of a supervisor, one male interviewer, three female interviewers, and one biomarker specialist. All the collected data, including data for antenatal care, institutional delivery and PNC, were checked for incompleteness, inconsistencies, and errors, and fieldworkers were informed to mitigate the problems. The NDHS used a standardized tool that has been validated across diverse global settings. In the context of Nepal, the tool underwent further refinement through expert consultations and had been previously employed in prior periodic surveys, establishing its reliability and validity. The details of data collection are mentioned in the study report [7].

### Dependent variables

We have three dependent variables in the study related to maternal health service use, which include (i) Four or more ANC visits (yes/no): the mother who received pregnancy care from skilled providers, such as doctors, nurses, and auxiliary nurse midwives for four times or more at a health facility were considered to have four or more ANC visits; (ii) Institutional delivery (yes/no): the condition where mother delivered her baby in health facilities was considered as institutional delivery, and (iii) PNC for mother and newborn (yes/no): the mother was considered to have PNC if both mother and newborn received care from care providers such as a doctor, nurses and midwives during first two days of child delivery.

### Independent variables

In this study, media exposure was the independent variable, which included exposure to mass media (radio, television, or newspaper), internet use and exposure to eight different specific health programs on television and radio. The women were considered to have mass media exposure if they reported reading newspapers, watching television, or listening to the radio at least once a week. The women were considered to have used the internet if they reported using the internet every day or at least once a week in the past month. Similarly, the women were considered to have had exposure to specific health programs if they heard or saw health programs on the radio or television sampled by the survey. These programs included eight different health-related programs broadcasted from radio and television namely *Jana Swasthya Bahas* (Public Health Debate) television program, *Jeevan Chakra* (Life Cycle) television serial, *Jana Swasthya* (Public Health) radio program, *Swasthya Gatibidhi* (Health Affairs) radio program, *Eak Dui Tin Sunau eekai Chhin* (Listen for a While) radio program, *Bhanchhin Ama* (Mother Says) radio program, *Hello Bhanchin Ama* (Mother Says Hello) radio program and *Jeewan Rakshya* (Save Life COVID- response) radio program.

### Adjusted variables

In the regression model, we adjusted for ecological belt (mountain, hill, Terai), setting (urban, rural), province (Koshi, Madhesh, Bagmati, Gandaki, Lumbini, Karnali, Sudurpashchim), age (in years), ethnicity (Brahmin or Chhetri, Dalit, Janajati, Madhesi, Other), religion (Hindu, non-Hindu), wealth quintile (poorest, poorer, middle, richer, richest), education (no education, basic (grade 1 to 8), secondary (grade 9–12), higher (above 12^th^ grade), occupation (not working, agriculture, professional or technical or manager or clerical, sales and service, skilled or unskilled labor, others).

### Statistical analysis

We analyzed data in R version 4.3.0 along with RStudio. A weighted analysis was performed using the "survey" package to account for the complex survey design of NDHS 2022. Categorical variables were presented as frequency, percentage, and 95% Confidence Interval (CI), while numerical variables were represented as median and interquartile range. We performed both univariate and multivariable logistic regression analyses to examine the association of mass media exposure, health program exposure and internet use with maternal health service use (including having at least 4 ANC visits, institutional delivery, and PNC). We performed a multicollinearity test and excluded variables with a variance inflation factor greater than five in the multivariable logistic regression model. The findings from the logistic regression analysis were presented as Crude Odds Ratio (COR) and Adjusted Odds Ratio (AOR) with their respective 95% CI.

### Ethical approval

We received approval from “The Demographic and Health Survey (DHS) program” upon our request to access and use the dataset for this study. The NDHS 2022 received ethical approval from the institutional review board of ICF International, United States of America (Reference number: 180657.0.001.NP.DHS.01, Date: 28^th^ April 2022) and the Ethical Review Board of Nepal Health Research Council (Reference number: 678, Date: 30^th^ September 2021). During the survey, written informed consent was obtained from all participants who were adults (18 years and above). For individuals below the age of 18, consent was obtained from their parents or guardians, and assent was obtained from the minor themselves.

## Results

Table 1 presents the characteristics of women aged 15–49 with live births in the two years before the survey and their exposure to different mass media, health programs and internet use. Out of 1,933 women, the majority were from urban areas 1,266 (65.5%) and the Terai Belt 1,166 (60.3%). The distribution of participants by province ranged from 117 (6.0%) in Gandaki province to 500 (25.8%) in Madhesh province. Similarly, 884 (45.7%) were exposed to mass media- radio, television, or newspapers, 1,041 (53.9%) used the internet, and 420 (21.8%) listened to or watched health programs on television or radio. 215 (11.1%) of participants were covered by health insurance schemes.

**Table 1:** Characteristics of participants (n = 1933).

Characteristics	n (%)
**Type of place of residence**	
Urban	1,266 (65.5%)
Rural	666 (34.5%)
**Ecological belt**	
Hill	639 (33.0%)
Mountain	129 (6.7%)
Terai	1,166 (60.3%)
**Province**	
Koshi	358 (18.5%)
Madhesh	500 (25.8%)
Bagmati	295 (15.3%)
Gandaki	117 (6.0%)
Lumbini	329 (17.0%)
Karnali	149 (7.7%)
Sudurpashchim	185 (9.6%)
**Ethnicity**	
Brahmin/Chhetri	499 (25.8%)
Dalit	359 (18.5%)
Janajati	588 (30.4%)
Madheshi	354 (18.3%)
Others	133 (6.9%)
**Religion**	
Hindu	1,611 (83.4%)
Non-Hindu	321 (16.6%)
**Age (in years** ^**) #**^	24.0 (21.0, 28.0)
< = 20 years	213 (11.0%)
21–34 years	1,616 (83.6%)
35–49 years	104 (5.4%)
**Age at childbirth**	
<20years	342 (17.7%)
20–34 years	1,516 (78.4%)
> = 35	75 (3.9%)
**Wealth index**	
Poorest	431 (22.3%)
Poorer	432 (22.3%)
Middle	381 (19.7%)
Richer	386 (20.0%)
Richest	303 (15.7%)
**Highest educational level**	
No education	357 (18.5%)
Basic	656 (34.0%)
Secondary	828 (42.9%)
Higher	91 (4.7%)
**Husband/partner’s education level**	
No education	183 (9.5%)
Basic	757 (39.5%)
Secondary	777 (40.5%)
Higher	163 (8.5%)
Don’t know	37 (1.9%)
Missing	15
**Occupation**	
Agriculture	866 (44.8%)
Not working	801 (41.4%)
Professional, managerial, technical and clerical workers	95 (4.9%)
Sales and service	74 (3.8%)
Skilled or unskilled labor	97 (5.0%)
**Husband/partner’s occupation**	
Agriculture	866 (44.8%)
Not working	801 (41.4%)
Professional, managerial, technical and clerical workers	95 (4.9%)
Sales and service	74 (3.8%)
Skilled or unskilled labor	97 (5.0%)
**Distance to health facility**	
> = 2 hours	68 (3.5%)
1 to 2 hour	152 (7.9%)
30–59 min	41 (2.1%)
Less than 30 min	1,671 (86.5%)
**Covered by health insurance**	215 (11.1%)
**Internet use**	1,041 (53.9%)
**Mass media exposure**	884 (45.7%)
**Health program exposure**	420 (21.8%)

# median (interquartile range)

Table 2 presents the study participants’ mass media exposure, internet use and health program exposure.

**Table 2:** Mass media, internet and health program exposure among women aged 15–49 with a live birth in the 2 years preceding the survey.

Characteristic	Mass Media exposure *	Internet use	Health program exposure
	n = 884	n = 1,041	n = 420
**Type of place of residence**			
Urban	625 (49.4%)	769 (60.7%)	253 (20.0%)
Rural	259 (38.8%)	273 (40.9%)	167 (25.1%)
**Ecological belt**			
Hill	335 (52.5%)	357 (56.0%)	207 (32.4%)
Mountain	63 (48.9%)	43 (33.5%)	68 (52.8%)
Terai	486 (41.7%)	641 (55.0%)	146 (12.5%)
**Province**			
Koshi	179 (50.2%)	173 (48.2%)	71 (20.0%)
Madhesh	161 (32.3%)	274 (54.9%)	23 (4.6%)
Bagmati	173 (58.5%)	200 (67.7%)	71 (24.1%)
Gandaki	61 (51.9%)	86 (73.8%)	29 (24.6%)
Lumbini	148 (44.9%)	194 (58.9%)	78 (23.8%)
Karnali	71 (47.4%)	45 (30.4%)	61 (40.8%)
Sudurpashchim	91 (49.2%)	69 (37.4%)	87 (46.9%)
**Ethnicity**			
Brahmin/Chhetri	290 (58.2%)	314 (62.9%)	204 (40.8%)
Dalit	124 (34.6%)	142 (39.6%)	63 (17.6%)
Janajati	303 (51.6%)	316 (53.8%)	138 (23.5%)
Madheshi	133 (37.6%)	189 (53.3%)	15 (4.3%)
Others	33 (24.9%)	81 (60.4%)	0 (0.0%)
**Religion**			
Hindu	761 (47.2%)	866 (53.8%)	381 (23.6%)
Non-Hindu	123 (38.2%)	175 (54.5%)	40 (12.4%)
**Age (in years)** ^**#**^	24.0 (22.0, 29.0)	24.0 (21.0, 28.0)	24.0 (22.0, 28.8)
< = 20 years	83 (39.0%)	107 (50.0%)	37 (17.3%)
21–34 years	757 (46.9%)	900 (55.7%)	358 (22.2%)
35–49 years	44 (42.0%)	35 (33.5%)	26 (24.6%)
**Age at childbirth**			
<20years	154 (45.2%)	176 (51.4%)	59 (17.4%)
20–34 years	700 (46.2%)	841 (55.5%)	343 (22.6%)
> = 35	29 (38.9%)	24 (32.4%)	18 (24.5%)
**Wealth index**			
Poorest	157 (36.3%)	112 (26.0%)	122 (28.3%)
Poorer	162 (37.5%)	175 (40.6%)	95 (22.0%)
Middle	175 (45.9%)	205 (53.8%)	81 (21.3%)
Richer	188 (48.7%)	281 (72.9%)	56 (14.6%)
Richest	203 (67.0%)	268 (88.4%)	66 (21.6%)
**Highest educational level**			
No education	68 (19.0%)	117 (32.8%)	18 (5.1%)
Basic	258 (39.4%)	280 (42.7%)	109 (16.6%)
Secondary	494 (59.7%)	558 (67.4%)	271 (32.7%)
Higher	64 (69.7%)	85 (93.4%)	22 (24.4%)
**Husband/partner’s education level**			
No education	33 (18.0%)	58 (31.6%)	11 (5.8%)
Basic	304 (40.1%)	334 (44.1%)	138 (18.2%)
Secondary	419 (53.9%)	489 (62.9%)	227 (29.3%)
Higher	109 (67.1%)	138 (84.4%)	44 (27.3%)
Don’t know	15 (41.8%)	14 (37.0%)	0 (0.0%)
Missing	3	9	0
**Occupation**			
Agriculture	389 (44.9%)	377 (43.5%)	234 (27.1%)
Not working	347 (43.3%)	477 (59.6%)	120 (15.0%)
Professional, managerial, technical and clerical workers	60 (63.6%)	87 (91.2%)	27 (28.0%)
Sales and service	43 (58.3%)	52 (70.2%)	20 (26.5%)
Skilled or unskilled labor	45 (46.1%)	48 (49.9%)	20 (20.5%)
**Husband/partner’s occupation**			
Agriculture	389 (44.9%)	377 (43.5%)	234 (27.1%)
Not working	347 (43.3%)	477 (59.6%)	120 (15.0%)
Professional, managerial, technical and clerical workers	60 (63.6%)	87 (91.2%)	27 (28.0%)
Sales and service	43 (58.3%)	52 (70.2%)	20 (26.5%)
Skilled or unskilled labor	45 (46.1%)	48 (49.9%)	20 (20.5%)
**Distance to HF**			
> = 2 hours	36 (52.3%)	14 (20.7%)	19 (27.3%)
1 to 2 hour	67 (44.1%)	52 (34.4%)	53 (34.9%)
30–59 min	12 (28.8%)	17 (40.0%)	10 (24.1%)
Less than 30 min	769 (46.0%)	958 (57.4%)	339 (20.3%)
**Covered by health insurance**	128 (59.3%)	157 (73.0%)	60 (27.8%)

# median (interquartile)

49.4% of women in urban and 38.8% of women in rural settings were exposed to mass media. Internet use (60.7%) and mass media use (49.4%) were higher among urban participants. Similarly, 60.7% of participants in urban and 40.9% in rural areas had used the internet, while the proportion of participants exposed to health programs was 20% in urban and 25.1% in rural settings. Among provinces, the exposure to mass media ranged from 32.2% in Madhesh to 58.5% in Bagmati. Internet use ranged from 30.4% in Karnali to 73.8% in Gandaki, while the exposure to health programs ranged from 4.6% in Madhesh to 46.9% in Sudurpashchim demonstrating variation across the provinces.

Breakdown by age found both mass media exposure and internet use were highest among women aged 21–34 (46.9% for mass media, 55.7% for internet). Mass media, internet use and health program exposure were highest among Brahmin/ Chhetri and Hindu women with professional, managerial, technical and clerical occupational backgrounds and women with the highest level of education. Mass media and internet exposure were lowest among poorest wealth quintile group.

**Fig 1:**
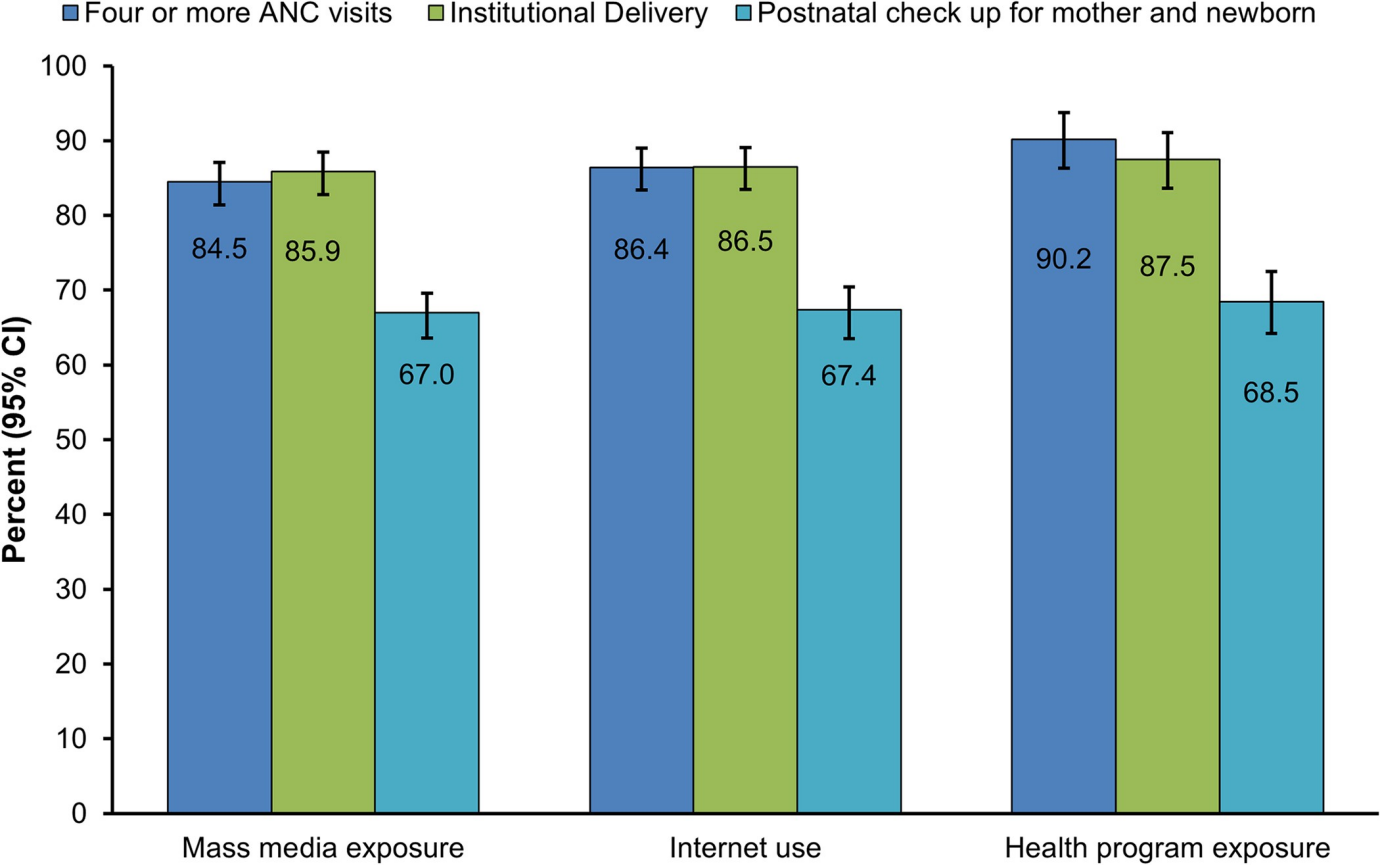
Maternal health service use by media exposure.

Fig 1 shows the proportion of maternal healthcare use by mass media exposure, internet use and health program exposure. Out of total of 884 participants exposed to mass media, 84.5% (95% CI:81.4, 87.1) completed four or more ANC visits, 85.9% (95% CI: 82.9, 88.5) had institutional delivery and 67.0% (95% CI: 72.0, 78.7) received PNC services. The women with four or more ANC visits, institutional delivery and PNC checkup were 86.4% (95% CI: 83.3, 89.0), 86.5% (95% CI: 83.5, 89.1) and 67.4% (95% CI: 73.0, 80.1) respectively among women who used the internet. Of a total of 420 women exposed to specific health programs on radio and television, 90.2% (95% CI: 86.8, 92.8) had four or more ANC visits, 87.5% (95% CI: 83.6, 90.5) had institutional delivery and 68.575.1% (95% CI: 71.0, 78.8) received PNC services (Fig 1).

Table 3 presents the association of media exposure with maternal health service use based on multivariable logistic regression analysis adjusted for ecological belt, place of residence, province, age, ethnicity, religion, marital status, wealth quintile, education, occupation, health insurance coverage and distance to health facility. The odds of having institutional delivery were 1.32 times higher (AOR: 1.32; 95% CI: 1.00, 1.74) among women with media exposure, while four or more ANC visits and PNC visits did not have statistically significant association. Participants having exposure to internet use had 1.59 times higher among participants using the internet (AOR: 1.59; 95% CI: 1.16, 2.19) of having four or more ANC visits. The participants exposed to health programs had 1.73 times higher odds of four or more ANC visits (AOR: 1.73; 95% CI: 1.17, 2.56) and 1.50 times higher odds of having institutional delivery (AOR = 1.50; 95% CI 1.02, 2.21) compared to their counterparts without the exposure.

**Table 3:** Association of mass media exposure, internet use and exposure to health programs with maternal health service use (ANC, ID, PNC).

Characteristic	Four or more ANC visits		Institutional Delivery		PNC checkup for mother and newborn	
	COR (95%CI)	AOR (95%CI)	COR (95%CI)	AOR (95%CI)	COR (95%CI)	AOR (95%CI)
**Mass media exposure**						
No	Ref	Ref	Ref	Ref	Ref	Ref
Yes	1.62 (1.23, 2.14)**	1.08 (0.80, 1.45)	2.14 (1.62, 2.83)**	1.32 (1.00, 1.74)*	1.41 (1.12, 1.78)**	1.13 (0.87, 1.46)
**Internet use**						
No	Ref	Ref	Ref	Ref	Ref	Ref
Yes	2.29 (1.70, 3.09)**	1.59 (1.16, 2.19)**	2.60 (1.98, 3.42)**	1.24 (0.92, 1.68)	1.55 (1.23, 1.95)**	1.05 (0.82, 1.35)
**Health program exposure**						
No	Ref	Ref	Ref	Ref	Ref	Ref
Yes	2.63 (1.79, 3.84)**	1.73 (1.17, 2.56)**	2.06 (1.48, 2.87)**	1.50 (1.02, 2.21)*	1.39 (1.12, 1.73)*	1.27 (0.99, 1.62)

Adjusted for place of residence, ecological belt, religion, age, wealth, education, husband education, occupation, health insurance coverage, distance to health facility

Ethnicity and province were excluded due to VIF>5 (collinearity)

COR: crude odds ratio; AOR: adjusted odds ratio; %: percent; Ref: Reference group

** indicates significance at 99% CI

* indicates significance at 95% CI

## Discussion

The results showed a diversified proportion of participants exposed to various types of media. The study indicated that over half of the women (53.9%) used the internet, while 45.7% were exposed to mass media like radio, television, or newspapers. A smaller proportion (21.8%) listened to or watched health programs on radio or television. This finding resonates with recent trends of mass media distribution in Nepal, with 76% of men and 65% of women being exposed to media in 2022 [7]. By province, the proportion of women exposed to mass media is highest in Bagmati province, which might be because of the high concertation of mass media in Bagmati, which also hosts the capital city, compared to other provinces [16].

Our study found that the use of maternal services was higher among the women who had been exposed to different forms of media, including mass media, the internet, and health programs, compared to those who had not been exposed to any form of media that echo the finding of previous studies in various countries in Asia and Africa [8–10]. A 2021 study that analyzed a dataset from nine countries between 2013 and 2017 found that watching television and reading newspapers had positive relationship with institutional delivery in sub-Saharan Africa [17]. Likewise, a study conducted in South Asian countries, including India, Bangladesh, Nepal, and Pakistan, identified a positive link between mass media exposure and the use of maternal healthcare services, such as an increased likelihood of giving birth in a healthcare facility [8, 18]. Comparable studies conducted in resource-limited settings in Africa, such as Ethiopia and Uganda, also highlighted a positive association between women’s exposure to television, radio and newspapers and their use of maternal health services. These media-exposed women were better informed [19, 20], highlighting the role of media in enhancing maternal healthcare uptake. This association reinforces the notion that the audience’s behaviors are influenced by the knowledge they receive from television, radio and newspaper and the way they understand the issues [19, 21].

Our findings further support prior research indicating a positive association between women’s internet use and having four or more ANC checkups. This adds weight to the idea that exposure to the internet increases the likelihood of improving maternal health service use [20, 22, 23] as the internet has emerged as a common source of information on maternal health [24]. These results correspond with the idea that maternal health information, accessible in household and hospital settings aided by internet and mobile devices, helps women to be better aware of health and better prepared for delivery services [25, 26]. Women may choose to take ANC services and have delivery in health facilities following high consumption of information during their pregnancy [27].

Additionally, our study revealed the association of exposure to health-related radio and television programs with ANC and institutional delivery. This indicates the possibility that health programs broadcasted from television and local radio stations targeting specific segments of women can lead to better use of maternal healthcare services [10]. Therefore, policymakers and program implementers may consider the media as an important stakeholder due to their role in increasing awareness and improving the use of maternal health services [24].

Our study did not show a positive association of all type of media with the PNC service use. This finding contrasts with other studies from South Asian countries and Ethiopia [8, 28–31]. This contrast may be due to differences in social and cultural settings among various countries. Exposure to mass media (radio, television, newspaper), internet use, and health programs on radio and television with maternal health services, were found to have impacted multiple maternal health service use variables. For instance, mass media exposure was associated with institutional delivery, the internet was associated with ANC, and listening to or watching health programs was associated with both ANC and institutional delivery. In the context of media convergence, it is also possible that media programs could have been accessed through the internet. However, further studies could reveal exposure to specific media are effective in some variables and not in others.

While the finding of our study points to the fact that media can serve as a potent tool to increase the uptake of maternal health services, there are other factors policymakers and program implementers could consider. Besides measures to increase women’s access to the internet and media tools, it is important to design contextual and audience-specific programs and focus on the strategic placement and dissemination of messages. This is because mass media’s effectiveness is determined by several factors like frequency of messages, degree of accuracy, audience’s suitability of the medium and health literacy of audience among other [21, 32].

### Strengths and limitations

Our findings can be useful particularly for policymakers, program designers and implementers to design health information, education and communication since this study is based on nationally representative data collected in a globally standardized tool and considers the federal structure during the sampling process and presents comparable findings. However, this study was not specifically designed to evaluate the media’s impact on maternal health service use and some important variables might have been missing such exposure to social media (Facebook, Instagram, Tiktok, Twitter), timing and duration of exposure to mass media, health programs, and internet use specific to maternal health. Nevertheless, these findings emphasize the opportunity to optimize media use in increasing awareness and maternal health services use and the urgency of focusing on the role of the media to enhance the uptake of maternal service use. Future studies could explore additional factors that intersect with media exposure, providing a more comprehensive understanding of how media can be helpful for the uptake of maternal health services such as ANC, Institutional Delivery and PNC.

## Conclusion

Our findings suggest that exposure to health programs and internet use is associated with increased ANC visits (four or more), while exposure to mass media and health programs is linked to an increased likelihood of opting for institutional delivery. These findings emphasize the need for media use to increase awareness and maternal health service use and the urgency of designing and implementing contextually tailored health-related media programs as an intervention to enhance maternal health outcomes in Nepal.

## References

[1] Acharya D, Khanal V, Singh JK, Adhikari M, Gautam S. Impact of mass media on the utilization of antenatal care services among women of rural community in Nepal. BMC Res Notes. 2015;8: 4–9. doi: 10.1186/s13104-015-1312-8 26264412 PMC4534014

[2] Department of Health Service. Annual Report. Kathmandu; 2022. Available: http://dohs.gov.np/wp-content/uploads/Annual_Report.pdf.

[3] Schwarz R, Thapa A, Sharma S, Kalaunee SP. At a crossroads: How can Nepal enhance its community health care system to achieve Sustainable Development Goal 3 and universal health coverage? J Glob Health. 2020;10: 1–4. doi: 10.7189/jogh.10.010309 32257137 PMC7100858

[4] Anwar A, Malik M, Raees V, Anwar A. Role of Mass Media and Public Health Communications in the COVID-19 Pandemic. Cureus. 2020;12. doi: 10.7759/cureus.10453 33072461 PMC7557800

[5] Karasneh R, Al-Azzam S, Muflih S, Soudah O, Hawamdeh S, Khader Y. Media’s effect on shaping knowledge, awareness risk perceptions and communication practices of pandemic COVID-19 among pharmacists. Res Soc Adm Pharm. 2021;17: 1897–1902. doi: 10.1016/j.sapharm.2020.04.027 32340892 PMC7179508

[6] Fombad MC, Jiyane GV. The role of community radios in information dissemination to rural women in South Africa. J Librariansh Inf Sci. 2019;51: 47–58. doi: 10.1177/0961000616668960

[7] Ministry of Health and Population. Nepal Demographic and Health Survey. Kathmandu; 2022.

[8] Fatema K, Lariscy JT. Mass media exposure and maternal healthcare utilization in South Asia. SSM—Popul Heal. 2020;11: 100614. doi: 10.1016/j.ssmph.2020.100614 32596437 PMC7306581

[9] Sohn M, Jung M. Effects of Empowerment and Media Use by Women of Childbearing Age on Maternal Health Care Utilization in Developing Countries of Southeast Asia. Int J Heal Serv. 2020;50: 32–43. doi: 10.1177/0020731419867532 31416404

[10] Zamawe COF, Banda M, Dube AN. The impact of a community driven mass media campaign on the utilisation of maternal health care services in rural Malawi. BMC Pregnancy Childbirth. 2016;16: 1–9. doi: 10.1186/s12884-016-0816-0 26819242 PMC4730729

[11] Wakefield MA, Loken B, Hornik RC. Use of mass media campaigns to change health behaviour. Lancet. 2010;376: 1261–1271. doi: 10.1016/S0140-6736(10)60809-4 20933263 PMC4248563

[12] Asp G, Pettersson KO, Sandberg J, Kabakyenga J, Agardh A. E 287,000. 2014;1: 1–10.

[13] Ministry of Foreign Affairs. Nepal Profile. 2022 [cited 6 Nov 2023]. Available: https://mofa.gov.np/about-nepal/nepal-profile/.

[14] Central Bureacy of Statistics Nepal. National Population and Housing Census 2021. Kathmandu; 2021. Available: https://censusnepal.cbs.gov.np/results/downloads/national.

[15] National Planning Commission of Nepal. Nepal Human Development Report 2020: Beyond Graduation. Kathmandu; 2020. Available: www.npc.gob.np.

[16] Ojha A, Kumar A. Mapping Nepal’s News Media Landscape: Different Dimensions and Emerging Issues. Asian Think. 2022;1296: 43–57.

[17] Gebremichael SG, Fenta SM. Determinants of institutional delivery in Sub-Saharan Africa: findings from Demographic and Health Survey (2013–2017) from nine countries. Trop Med Health. 2021;49. doi: 10.1186/s41182-021-00335-x 34039443 PMC8152346

[18] Kabir MR. How do traditional media access and mobile phone use affect maternal healthcare service use in Bangladesh? Moderated mediation effects of socioeconomic factors. PLoS One. 2022;17: 1–17. doi: 10.1371/journal.pone.0266631 35476825 PMC9045672

[19] Seidu AA, Ahinkorah BO, Hagan JE, Ameyaw EK, Abodey E, Odoi A, et al. Mass Media Exposure and Women’s Household Decision-Making Capacity in 30 Sub-Saharan African Countries: Analysis of Demographic and Health Surveys. Front Psychol. 2020;11: 1–11. doi: 10.3389/fpsyg.2020.581614 33192898 PMC7655773

[20] Dewau R, Muche A, Fentaw Z, Yalew M, Bitew G, Amsalu ET, et al. Time to initiation of antenatal care and its predictors among pregnant women in Ethiopia: Cox-gamma shared frailty model. PLoS One. 2021;16: 1–18. doi: 10.1371/journal.pone.0246349 33544714 PMC7864666

[21] Westwood B, Westwood G. Assessment of newspaper reporting of public health and the medical model: A methodological case study. Health Promot Int. 1999;14: 53–64. doi: 10.1093/heapro/14.1.53

[22] Igbinoba AO, Soola EO, Omojola O, Odukoya J, Adekeye O, Salau OP. Women’s mass media exposure and maternal health awareness in Ota, Nigeria. Cogent Soc Sci. 2020;6. doi: 10.1080/23311886.2020.1766260

[23] Wang Y, Etowa J, Ghose B, Tang S, Ji L, Huang R. Association between mass media use and maternal healthcare service utilisation in malawi. J Multidiscip Healthc. 2021;14: 1159–1167. doi: 10.2147/JMDH.S304078 34045863 PMC8144173

[24] Ohaja M, Senkyire EK, Ewetan O, Asiedua E, Azuh D. A narrative literature review on media and maternal health in Africa. World Med Heal Policy. 2023;15: 123–147. doi: 10.1002/WMH3.546

[25] Schnitman G, Wang T, Kundu S, Turkdogan S, Gotlieb R, How J, et al. The role of digital patient education in maternal health: A systematic review. Patient Educ Couns. 2022;105: 586–593. doi: 10.1016/j.pec.2021.06.019 34183217

[26] Chae JM, Kim HK. Internet-based prenatal interventions for maternal health among pregnant women: A systematic review and meta-analysis. Child Youth Serv Rev. 2021;127: 106079. doi: 10.1016/j.childyouth.2021.106079

[27] Narasimhulu DM, Karakash S, Weedon J, Minkoff H. Patterns of Internet Use by Pregnant Women, and Reliability of Pregnancy-Related Searches. Matern Child Health J. 2016;20: 2502–2509. doi: 10.1007/s10995-016-2075-0 27456311

[28] Islam MR, Odland JO. Determinants of antenatal and postnatal care visits among indigenous people in Bangladesh: A study of the Mru community. Rural Remote Health. 2011;11: 1–13. doi: 10.22605/rrh1672 21714582

[29] Regassa N. Antenatal and postnatal care service utilization in Southern Ethiopia: A population-based study. Afr Health Sci. 2011;11: 390–397. 22275929 PMC3260999

[30] Angore BN, Tufa EG, Bisetegen FS. Determinants of postnatal care utilization in urban community among women in Debre Birhan Town, Northern Shewa, Ethiopia. J Heal Popul Nutr. 2018;37: 1–9. doi: 10.1186/s41043-018-0140-6 29673402 PMC5909206

[31] Paul P, Chouhan P. Socio-demographic factors influencing utilization of maternal health care services in India. Clin Epidemiol Glob Heal. 2020;8: 666–670. doi: 10.1016/j.cegh.2019.12.023

[32] Ngigi S, Busolo DN. Behaviour Change Communication in Health Promotion: Appropriate Practices and Promising Approaches. Int J Innov Res Dev. 2018;7. doi: 10.24940/ijird/2018/v7/i9/sep18027

